# Visualisation of epidemiological map using an Internet of Things infectious disease surveillance platform

**DOI:** 10.1186/s13054-020-03132-w

**Published:** 2020-07-09

**Authors:** Guanghao Sun, Nguyen Vu Trung, Le Thi Hoi, Pham Thanh Hiep, Koichiro Ishibashi, Takemi Matsui

**Affiliations:** 1 Graduate School of Informatics and Engineering, The University of Electro-Communications, 1-5-1 Chofugaoka, Chofu, Tokyo 182-8585 Japan; 2 National Hospital for Tropical Diseases, Hanoi, Vietnam; 3 Hanoi Medical University, Hanoi, Vietnam; 4 Le Quy Don Technical University, Hanoi, Vietnam; 5 Tokyo Metropolitan University, Tokyo, Japan

**Keywords:** Mass screening, Internet of Things, Infection, Surveillance

Dear Editor,

We read with the interest Editorial by Verdonk et al. on how machine learning could be used in clinical practice during an epidemic such as the coronavirus disease 2019 (COVID-19) [1]. The COVID-19 pandemic has resulted in a global public health emergency. Border control measures such as symptom screening and health questionnaires have been enforced at many international airports [2]. However, recent research and experiences show that many infected travellers can slip through fever-based screening which employs thermography, due to false-negative results [3].

Vital signs, especially the body temperature, are the most frequent symptoms for infectious diseases. We have developed a novel non-contact vital sign measurement system for screening infectious diseases; this system can detect suspected infections based on contactless multiple vital sign monitoring, thereby outperforming fever screening [4]. The most promising approach towards improving the performance involves connecting multiple systems to an Internet of Things (IoT) infectious disease surveillance platform. This could enable operators to predict the outbreaks of infectious diseases earlier than is currently possible.

The concept of IoT infectious disease surveillance platform is presented in Fig. 1. The targeted vital signs are heart rate, respiration rate, and body temperature, which can be measured without contact by using the infectious diseases screening radar system designed for airport quarantine counter and onboard screening (Fig. 1a, b). Moreover, data on ambient temperature, humidity, and global positioning system (GPS) can be simultaneously monitored. The server displays these values according to the IP and GPS information of each system and records these values in addition to the original vital signals and thermal images in its big database, in association with the patient’s ID for future analyses (Fig. 1c). We are investigating methods of improvement and potential novel applications towards the visualisation of the epidemiological map (Fig. 1e, f) via the combination of big data analysis and artificial neural network. For instance, standalone infection screening systems can be placed in airport quarantines, outpatient units, and other places across Japan where mass gatherings are likely to occur. The IoT infectious disease surveillance platform interconnects all infection screening systems, facilitating the collection and transmission of big data via internet. These data can be comprehensively analysed to conduct real-time surveillance and visualisation of the epidemiological map and thereby capture hot-spots or clusters of cases. This technology will enable timely tracking of epidemic outbreaks and facilitate faster decision-making which can delay exponential increase in the number of infected cases (Fig. 1g).

**Fig. 1:**
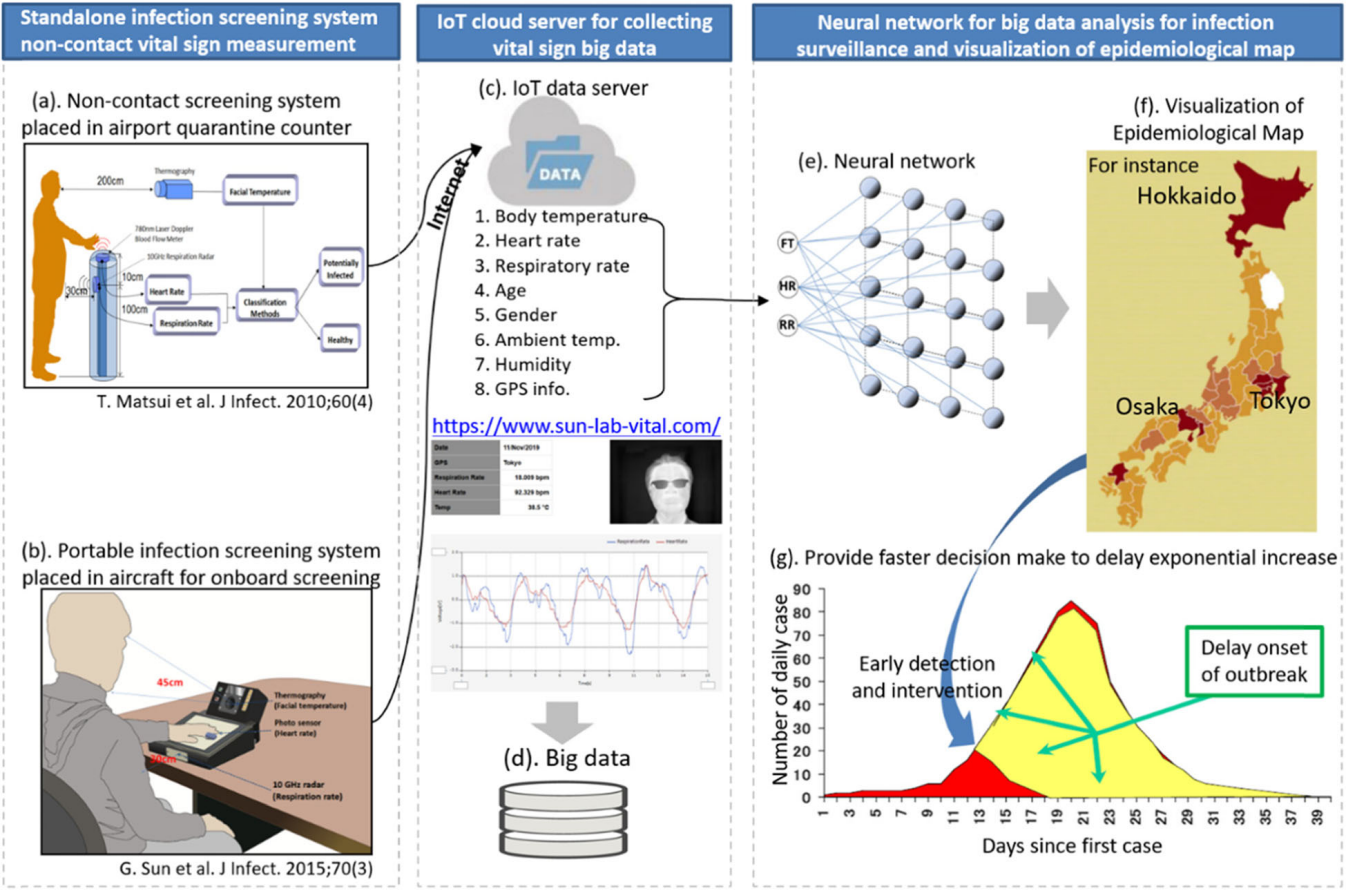
Potential network structure of a vital signs-based infection screening system used for the early detection and prediction of pandemic outbreak of infectious diseases

Advancements in innovative technologies, such as IoT, AI, 5G, and big data are leading to the emergence of a new field in the fight against COVID-19 and other pandemics. Integration of such technologies can help to generate solutions within the healthcare sector for the screening, prediction, and prevention of emerging infectious diseases.

## Data Availability

Not applicable.
